# Supplementary material for: Evaluation of the Stability of Bacteriophages in Different Solutions Suitable for the Production of Magistral Preparations in Belgium
Source: Viruses. 2021 May 8;13(5):865. doi: 10.3390/v13050865 (PMC8151234; doi:10.3390/v13050865)
Supplement: Supplementary file 1 [file viruses-13-00865-s001.zip › viruses-1192320-supplement.pdf]

viruses

MDPI

Article

Evaluation of the stability of bacteriophages in different solutions suitable for the production of magistral preparations in Belgium

Hans Duyvejonck [1,2], Maya Merabishvili [2,3], Mario Vaneechoutte [2], Steven de Soir [3], Rosanna Wright [4,5], Ville-Petri Friman [4], Gilbert Verbeken [3], Daniel De Vos [3], Jean-Paul Pirnay [3], Els Van Mechelen [1] and Stefan J. T. Vermeulen [1,*]

[1] Research Center Health & Water Technology, University College Ghent, Keramiekstraat 80, B-9000 Gent, Belgium; Els.vanmechelen@hogent.be (E.V.M.); stefan.vermeulen@hogent.be (S.J.T.V)

[2] Laboratory Bacteriology Research, Faculty of Medicine & Health Sciences, Ghent University, C. Heymanslaan 10, B-9000 Gent, Belgium; mario.vaneechoutte@ugent.be (M.V.)

[3] Laboratory for Molecular and Cellular Technology, Queen Astrid Military Hospital, Bruynstraat 1, 1120, Brussel, Belgium; maia.merabishvili@mil.be (M.M.); steven.desoir@mil.be (S.D.S.); Gilbert.Verbeken@mil.be (G.V.); DanielMarie.DeVos@mil.be (D.D.V.); jean-paul.pirnay@mil.be (J.-P.P)

[4] Department of Biology, University of York, Wentworth Way, York, YO10 5DD, UK; vifriman@gmail.com (V.F.)

[5] Division of Evolution and Genomic Sciences, University of Manchester, Dover Street, Manchester, M13 9PT, UK; rosanna.wright-2@manchester.ac.uk (R.W.)

* Correspondence: e-mail: stefan.vermeulen@hogent.be (S.J.T.V); 0032 498496997, Research Center Health & Water Technology, University College Ghent, Keramiekstraat 80, B-9000 Gent, Belgium

Table S1: Stability over time of the infectivity of different phages, expressed as log pfu/ml stored at 4 °C at two different starting concentrations: 7 log pfu/ml and 9 log pfu/ml.

Phage	Storage solution	Presumed titre pfu/ml	Phage titers (log pfu/ml) after storage period (days) 0	1	3	7	10	14	21	28	34	43	56	64	243	286	554
Acibel 004	5% Glucose	9 log	8.89	8.01	7.43	6.94	NT	6.62	NT	NT	6.69	6.73	NT	6.66	NT	NT	NT
		7 log	5.97	3.31	NT	0*	NA	NA	NA	NA	NA	NA	NA	NA	NA	NA	NA
	0.9 % NaCl	9 log	9.37	9.36	9.25	8.92	8.87	8.61	9.36	9.2	9.23	NT	NT	NT	NT	NT	8.99
		7 log	7.72	7.71	7.48	7.08	6.98	6.62	6.2	5.74	5.57	NT	NT	NT	NT	NT	0*
	Hartmann's infusion solution	9 log	9.45	9.46	9.31	9.22	9.16	9.15	9.33	9.15	9.22	NT	NT	NT	NT	NT	9.01
		7 log	7.79	7.7	7.45	7.11	7.08	6.76	6.28	5.9	5.7	NT	NT	NT	NT	NT	1.44
	DPBS w/ $CaMg^{2+}$	9 log	9.46	9.43	9.33	9.38	9.3	9.24	9.35	9.22	9.31	NT	NT	NT	NT	NT	9.03
		7 log	7.8	7.84	7.47	7.16	7.08	6.83	6.39	5.99	5.76	NT	NT	NT	NT	NT	1.33
	DPBS w/o $CaMg^{2+}$	9 log	9.44	9.43	9.36	9.38	9.35	9.26	9.33	9.19	9.29	NT	NT	NT	NT	NT	8.95
		7 log	7.62	7.67	7.39	7.08	7.01	6.75	6.41	5.91	5.67	NT	NT	NT	NT	NT	1.83
PNM	5% Glucose	9 log	0*	NA	NA	NA	NA	NA	NA	NA	NA	NA	NA	NA	NA	NA	NA
		7 log	0*	NA	NA	NA	NA	NA	NA	NA	NA	NA	NA	NA	NA	NA	NA
	0.9 % NaCl	9 log	9.20	9.65	9.28	9.16	8.56	9.43	9.18	NT	9.20	NT	NT	NT	NT	7.95	NT
		7 log	7.28	7.09	6.56	6.70	6.08	6.03	5.39	NT	3.80	NT	NT	NT	NT	NT	NT
	Hartmann's infusion solution	9 log	9.40	9.18	9.00	9.15	8.47	8.84	8.79	NT	9.01	NT	NT	NT	NT	7.05	NT
		7 log	7.55	6.95	6.94	6.72	6.21	5.89	5.22	NT	3.52	NT	NT	NT	NT	NT	NT
	DPBS w/ $CaMg^{2+}$	9 log	9.52	9.14	9.20	9.10	8.95	9.02	8.89	NT	8.97	NT	NT	NT	NT	9.06	NT
		7 log	7.41	7.15	6.93	6.63	6.53	5.88	5.28	NT	3.84	NT	NT	NT	NT	NT	NT
	DPBS w/o $CaMg^{2+}$	9 log	9.04	9.13	9.34	9.43	9.33	9.46	9.14	NT	9.18	NT	NT	NT	NT	9.36	NT
		7 log	7.18	7.17	6.62	6.50	6.70	6.08	5.38	NT	4.33	NT	NT	NT	NT	NT	NT
14/1	5% Glucose	9 log	9.83	8.91	NT	NT	NT	7.75	NT	3.60	NT	NT	2.24	NT	0*	NT	NT
		7 log	7.41	6.76	NT	NT	NT	4.53	NT	2.09	NT	NT	0*	NA	NA	NA	NA
	0.9 % NaCl	9 log	9.80	9.21	NT	NT	NT	9.27	NT	8.62	NT	NT	8.39	NT	7.69	NT	NT
		7 log	7.32	6.92	NT	NT	NT	7.10	NT	6.44	NT	NT	5.28	NT	2.39	NT	NT
	Hartmann's infusion solution	9 log	9.84	9.22	NT	NT	NT	8.27	NT	8.59	NT	NT	8.13	NT	7.57	NT	NT
		7 log	7.14	7.13	NT	NT	NT	7.14	NT	6.40	NT	NT	5.41	NT	2.14	NT	NT
	DPBS w/ $CaMg^{2+}$	9 log	9.41	9.07	NT	NT	NT	9.26	NT	8.70	NT	NT	8.39	NT	8.38	NT	NT
		7 log	7.28	7.02	NT	NT	NT	7.40	NT	6.44	NT	NT	4.76	NT	1.49	NT	NT
	DPBS w/o $CaMg^{2+}$	9 log	9.45	9.13	NT	NT	NT	9.43	NT	8.20	NT	NT	8.36	NT	8.79	NT	NT
		7 log	7.33	7.28	NT	NT	NT	6.86	NT	6.49	NT	NT	5.48	NT	2.70	NT	NT
ISP**	5% Glucose	8 log	0*	NA	NA	NA	NA	NA	NA	NA	NA	NA	NA	NA	NA	NA	NA
		7 log	0*	NA	NA	NA	NA	NA	NA	NA	NA	NA	NA	NA	NA	NA	NA
	0.9 % NaCl	8 log	8.76	8.71	8.60	8.64	8.54	8.62	8.62	NT	8.06	NT	NT	NT	NT	7.60	NT
		7 log	7.60	7.34	7.30	7.10	7.11	6.74	6.09	NT	5.19	NT	NT	NT	NT	NT	NT

**Hartmann's**	8 log	8.80	8.63	8.46	8.62	8.53	8.59	8.63	NT	8.04	NT	NT	NT	NT	7.40	NT
**infusion solution**	7 log	7.99	7.30	7.30	7.07	7.32	6.79	6.48	NT	4.84	NT	NT	NT	NT	NT	NT
**DPBS w/**	8 log	8.63	8.59	8.61	8.77	8.65	8.73	8.64	NT	8.18	NT	NT	NT	NT	7.89	NT
**$CaMg^{2+}$**	7 log	7.60	7.59	7.41	7.01	7.31	6.88	6.50	NT	4.95	NT	NT	NT	NT	NT	NT
**DPBS w/o**	8 log	8.62	8.47	8.71	8.97	8.32	8.87	8.88	NT	8.34	NT	NT	NT	NT	8.11	NT
**$CaMg^{2+}$**	7 log	7.63	7.18	7.37	7.08	7.08	6.81	6.51	NT	6.03	NT	NT	NT	NT	NT	NT

*, no plaques were observed in non-diluted sample, so titer could not be expressed in logs; * *, 8.8-8.9 log pfu/ml for phage ISP, NT, not tested; NA, not applicable.

Table S2: Stability of ISP phage (9 log pfu/ml) in two different stabilizers after freeze-drying procedure and storage at 4°C.

stabilizers	Before lyophilization	After lyophilization	After 3 months	After 12 months	After 17 months	After 27 months	After 8 years	After 126 days of resuspension of 8 years old samples
Sucrose 0.3M	9.33	7.93	7.93	7.94	7.90	7.92	7.99	7.72
Sucrose 0.5M	9.35	8.72	8.66	8.66	8.65	8.40	8.12	8.09
Sucrose 0.8M	9.35	8.92	8.84	8.64	8.63	8.62	7.73	8.39
Sucrose 1.0M	9.36	8.85	8.81	8.71	8.71	8.69	8.55	8.31
Trehalose 0.3M	9.35	8.69	8.61	8.61	8.56	7.68	7.24	6.80
Trehalose 0.5M	9.34	8.64	8.60	8.62	8.62	8.36	7.60	7.24
Trehalose 0.8M	9.35	8.72	8.72	8.71	8.69	8.65	6.92	6.41
Trehalose 1.0M	9.37	8.75	8.75	8.72	8.71	8.70	8.30	8.21

Table S3: Stability of resuspended ISP lyophilizates after storage at 4°C.

Stabilizer	concentration	Log pfu/ml	
		Days after resuspension	
		0	126
Sucrose	0.3 M	8.02	7.72
	0.5 M	8.03	8.09
	0.8 M	8.75	8.39
	1 M	8.59	8.31
Trehalose	0.3 M	7.10	6.80
	0.5 M	7.61	7.24
	0.8 M	6.90	6.41
	1 M	8.30	8.21
